# Longitudinal predictors of health-related quality of life in isolated dystonia

**DOI:** 10.1007/s00415-023-12022-4

**Published:** 2023-10-15

**Authors:** Johanna Junker, James Hall, Brian D. Berman, Marie Vidailhet, Emmanuel Roze, Tobias Bäumer, Irene A. Malaty, Aparna Wagle Shukla, Joseph Jankovic, Stephen G. Reich, Alberto J. Espay, Kevin R. Duque, Neepa Patel, Joel S. Perlmutter, H. A. Jinnah, Dystonia Coalition Study Group, Valerie Brandt, Norbert Brüggemann

**Affiliations:** 1 Department of Neurology, University of Luebeck, Ratzeburger Allee 160, 23538 Lübeck, SH Germany; 2 Institute of Neurogenetics, University of Luebeck, Luebeck, Germany; 3 Southampton Education School, University of Southampton, Southampton, UK; 4 Department of Neurology, Virginia Commonwealth University, Richmond, VA USA; 5 Departement de Neurologie, AP-HP, Hopital de La Pitie-Salpetriere, Paris, France; 6 Institut du Cerveau_ Paris Brain Institute-ICM, INSERM 1127, CNRS 7225, Sorbonne Université, Paris, France; 7 Institute of Systems Motor Science, University of Luebeck, Luebeck, Germany; 8 Department of Neurology, Fixel Institute for Neurologic Disorders, University of Florida, Gainesville, FL USA; 9 Parkinson’s Disease Center and Movement Disorders Clinic, Department of Neurology, Baylor College of Medicine, Houston, TX USA; 10 Department of Neurology, School of Medicine, University of Maryland, Baltimore, MD USA; 11 Department of Neurology, University of Cincinnati, Cincinnati, OH USA; 12 RUSH Parkinson’s Disease and Movement Disorders Center, Department of Neurological Science, RUSH University Medical Center Chicago, Chicago, IL USA; 13 Departments of Neurology, Radiology and Neuroscience, Washington University in St. Louis, St. Louis, MO USA; 14 Department of Neurology and Human Genetics, Emory University, Atlanta, GA USA; 15 School of Psychology, Centre for Innovation in Mental Health, University of Southampton, Southampton, UK

**Keywords:** Dystonia, Quality of life, Depression, Anxiety, BoNT

## Abstract

**Objective:**

To determine longitudinal predictors of health-related quality of life (HR-QoL) in an international multicenter cohort of patients with isolated dystonia.

**Methods:**

Out of 603 dystonia patients prospectively enrolled in the Natural History Dystonia Coalition study, 155 were assessed three times within 2 years for HR-QoL, symptoms of depression, generalized anxiety disorder (GAD), and social anxiety disorder (SAD), as well as dystonia severity and dystonic tremor. In addition, the impact of botulinum neurotoxin (BoNT) injections on HR-QoL was evaluated after 1 year.

**Results:**

Depressive symptoms at baseline predicted lower HR-QoL on all subscales after 2 years (all *p* ≤ 0.001). Higher GAD scores at baseline predicted lower HR-QoL related to general health, pain and emotional well-being, whereas higher SAD scores predicted higher pain-related QoL after 2 years (all *p* ≤ 0.006). Dystonia severity at baseline predicted social functioning (*p* = 0.002). Neither dystonic tremor, age, or sex predicted HR-QoL at 2 years. Two latent categories were revealed across the three-time points: Category 1 with higher total HR-QoL scores (mean HR-QoL = 74.4% ± 16.1), susceptible to symptoms of depression and SAD, and Category 2 with lower total HR-QoL scores (mean HR-QoL = 45.5% ± 17.6), susceptible to symptoms of GAD. HR-QoL improved over the course of 1 year irrespective of the use of BoNT.

**Conclusion:**

The longitudinal impact of psychiatric symptoms on HR-QoL emphasizes the importance of incorporating mental health treatment, in particular also the therapy of anxiety disorders, into treatment regimens for dystonia.

**Supplementary Information:**

The online version contains supplementary material available at 10.1007/s00415-023-12022-4.

## Introduction

Nonmotor symptoms (NMS), i.e. depression, generalized anxiety disorder (GAD), social anxiety disorder (SAD), self-esteem and acceptance of illness often concomitate the motor manifestations in isolated dystonia, occur more frequently than in healthy controls [1–7] and impact on the health-related quality of life (HR-QoL) [8–12]. The previous studies on HR-QoL in dystonia have been cross-sectional [8–14] and do not allow conclusions on predictors of future QoL. Longitudinal examinations in cranial and cervical dystonia several weeks after botulinum neurotoxin (BoNT) injection revealed an improvement of dystonia severity and improvement of certain HR-QoL domains [3, 15–20]. The prevalence of NMS such as depression and anxiety and the reported QoL in a small group of patients with cervical dystonia did, however, not change within 2 years [21].

This is the first study analyzing HR-QoL data in a large, international, prospective cohort of patients with adult-onset isolated dystonia to identify predictors of HR-QoL at 2 years after baseline examination. As BoNT is an established therapy to alleviate the severity of MS in dystonia, we additionally evaluated the longitudinal effects of BoNT on HR-QoL. Identifying predictors of HR-QoL in dystonia can help clinicians screen for patients at high risk of low HR-QoL and develop long-term therapy regimens.

## Methods

### Participants

Data from the Natural History Project of the Dystonia Coalition enrolled across 36 clinical sites (USA, Canada, Australia, Germany, France, Italy) were analyzed. Additional Dystonia Coalition investigators that contributed subjects to the study are listed in Online Resource 1. The multicenter Dystonia Coalition study includes dystonia patients aged 18 years and older, and the Natural History Project focuses on patients with dystonia onset no more than 5 years before study enrollment (https://www.rarediseasesnetwork.org/cms/dystonia).

Participants included in this study were examined three times within 2 years (baseline, 1-year follow-up, 2-year follow-up). At each study visit, the subjects answered standardized questionnaires and were clinically examined using a standardized protocol. Dystonia patients receiving BoNT treatment were enrolled when symptoms returned. This usually meant they were enrolled 3 months after treatment, but never sooner than two months after treatment. Exclusion criteria were secondary or combined dystonia and medical/neurological conditions confounding diagnoses or precluding a complete assessment.

### Questionnaire and rating scales

Information regarding the questionnaire and rating scales (Dystonia Coalition Questionnaire, RAND 36-Item Health Survey, Hospital Anxiety and Depression Scale (HADS), Liebowitz Social Anxiety Scale (LSAS)), as well as the examination protocol and rating (Burke − Fahn − Marsden Dystonia Rating Scale (BFMDRS), tremor) have been previously published [8].

HR-QoL was evaluated by the eight subscales (general health, physical functioning, role limitations due to physical health problems, pain, energy/fatigue, emotional well-being, role limitations due to emotional problems and social functioning) and the total score of the RAND 36-Item Health Survey [22]. Total HR-QoL is represented by the mean of the eight subscales of the RAND 36-Item Health Survey. The severity of generalized anxiety and depressive symptoms was assessed by the self-reported HADS version 4 [23] and symptoms of social anxiety by the LSAS [24]. The clinical examination included the evaluation of dystonia severity, using the validated BFMDRS [25], as well as the existence of tremors (yes/no).

### Statistical analysis

Predictors of 2-year HR-QoL subdomains were obtained from eight cross-lagged path models (CLPM, one model per domain; Bonferroni corrected alpha ≤ 0.006). Further, an independent analysis was done to categorize patients into clusters of high and low total HR-QoL within the 2 years period using a latent class growth analysis (LCGA). Data at baseline included 603 patients. Only patients with complete data sets at time point three (2-year follow-up) were included in the CLPM (*n* = 155) and only patients with complete data sets across the three time points (baseline, 1-year follow-up, and 2-year follow-up) were included in the LCGA (*n* = 145). Both the CLPM and the LCGA were carried out in MPlus version 7.4 [26].

**Fig. 1:**
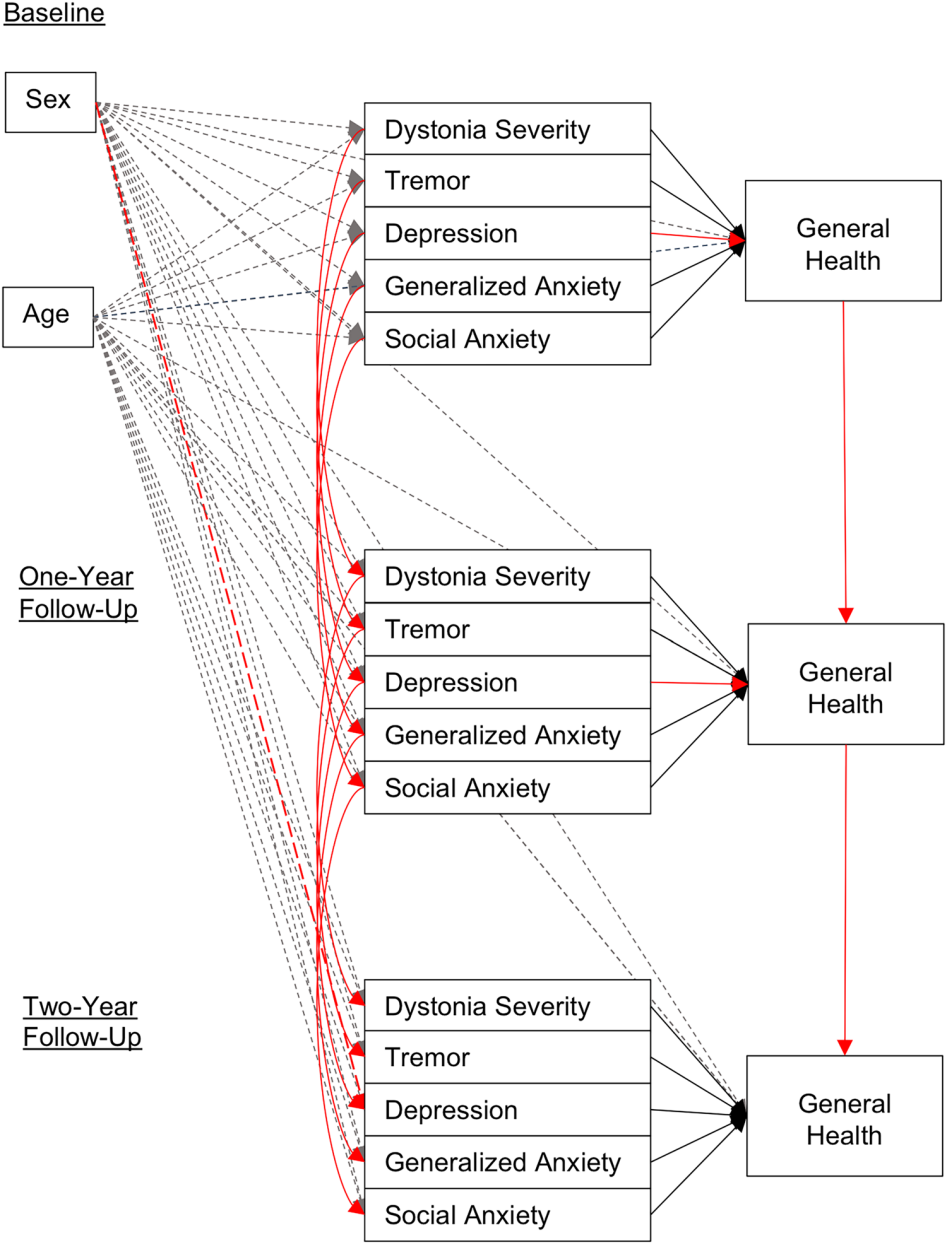
Direct effects of the cross-lagged path model for the general health subscale (stylized illustration). Direct paths of the cross-lagged path model for the general health subscale of HR-QoL are presented. Dashed lines illustrate the effect of age and sex on the different variables. Bonferroni corrected alpha is ≤ 0.006. Significant paths are marked in red, grey paths did not reach significance level. The other seven HR-QoL cross-lagged path models are presented in Online Resource 2_Fig. 3

Within the CLPM, the statistical effects of baseline dystonia severity, tremor (yes/no), age, sex, and symptom severity of depression (HADS-D), GAD (HADS-A) and SAD (LSAS), as well as the eight subscales of the RAND 36-Item Health Survey, were assessed as direct paths from baseline on the same variables at 1-year follow-up and as indirect paths via (intermediate) direct effects on the same variables at 2-year follow up (Fig. 1 and Online Resource 2_Fig. 3). The eight cross-lagged path models assessed the strength of directional associations between constructs over time while controlling for all other constructs included in the model and the correlations between the concepts at each time point [27]. Model fit was evaluated based on the proportions of variation (*r*^2^) that were explained in each statistically dependent variable [28].

To investigate the impact of dystonia severity, sex, age, the severity of depression, GAD and SAD on the development of total quality of life over time, linear growth in HR-QoL was estimated over the 2 years and combined with a statistical examination for distinct patterns of growth (‘latent classes’; hereafter, ‘categories’ to ease interpretation by clinicians). Robust maximum likelihood estimation was used to minimize biased results caused by non-normally distributed variables. An increasing number of categories were investigated, and the accuracy of the resulting categories was subjected to two formal statistical tests (Lo-Mendell − Rubin adjusted likelihood ratio test (LMR), Vuong − Lo-Mendell − Rubin likelihood ratio test (VLMR)) as to whether a statistical model estimating n categories offered a more accurate representation of the data than the n-1 category alternative. A two-category solution returned a significantly more accurate representation of the data than did a single category alternative (LMR: *p* = 0.042, VLMR: *p* = 0.041). However, there was also no significant improvement in the model fit by extending the estimate to three categories (LMR: *p* = 0.217, VLMR: *p* = 0.214). Furthermore, the two-category model was better able to represent HR-QoL over the three measurement points than was the less parsimonious three-category alternative (entropy value of 0.92 as compared to 0.87).

Annual changes of HR-QoL in dystonia were evaluated using a nonparametric ANalysis Of VAriance (ANOVA). Only patients with complete longitudinal data sets across the two time points were included in the ANOVA (*n* = 72). Data from two subgroups of patients were analyzed: patients not treated with BoNT (neither at baseline nor at follow-up, *n* = 37) and patients who received BoNT within 1-year of follow-up but not before baseline (n = 35). The analysis was corrected for oral medication intake such that only patients with unchanged or no oral medication at both time points were considered. Categorization of dystonia medication was performed according to Richardson et al.[29]. For this ANOVA, patients with local surgical treatment for dystonia (myectomy, denervation) were excluded (*n* = 3). The analysis was performed in R version 1.1.463. Mann − Whitney and Chi-square tests were run in SPSS Statistics 22 (IBM Corp., Armonk, NY) to evaluate group differences. All tests of significance were two-sided. *P* values ≤ 0.05 were considered significant unless Bonferroni-corrected.

### Data availability

Anonymized data (study protocol, statistical analysis) will be shared by request from any qualified investigator. Data will be available for 10 years.

### Results

Out of 603 participants, complete longitudinal data sets for the CLPM and the LCGA were available from 155 patients (representing 25.7% from our previous study [8]). The majority of patients were female (63.9%, 99/155), the mean age at baseline examination was 57.6 ± 11.1 years, and the mean age at dystonia onset was 54.8 ± 10.9 years. Table 1 displays mean BFMDRS scores, the ratio of patients with tremor, mean of RAND 36-Item Health Survey scores, HADS-D, HADS-A, and LSAS, as well as the ratio of patients with symptoms of depression (HADS-D > 7), GAD (HADS-A > 7), and SAD (LSAS > 30) at baseline.

**Table 1:** Clinical data of the Natural History Dystonia Coalition Study cohort at baseline

	HR-QoL	Depression	Generalized Anxiety	Social Anxiety	BFMDRS	Tremor
All (*n* = 155)	65.6 ± 21.3	26.5% (41/ 155) 4.9 ± 4.3	39.4% (61/155) 6.7 ± 3.9	45.2% (70/155) 32.9 ± 25.4	7.1 ± 5.9	41.9% (65/155)
Main groups of Dystonia						
focal (*n* = 93)	70.8 ± 19.5	22.6% (21/93) 4.3 ± 4.0	43.0% (40/93) 6.5 ± 3.8	36.6% (34/93) 28.5 ± 23.9	4.3 ± 2.2	43.0% (40/93)
Segmental (*n* = 49)	57.0 ± 21.1	32.7% (16/49) 6.1 ± 4.7	30.6% (15/49) 7.2 ± 4.1	67.3% (33/49) 42.6 ± 24.4	10.0 ± 6.0	32.7% (16/49)
Multifocal (*n* = 10)	60.3 ± 23.1	30.0% (3/10) 4.6 ± 3.8	40.0% (4/10) 6.1 ± 3.3	20.0% (2/10) 22.9 ± 19.7	10.1 ± 3.6	70.0% (7/10)
Generalized (*n* = 3)	62.2 ± 34.6	33.3% (1/3) 5.0 ± 4.6	66.7% (2/3) 9.3 ± 6.4	33.3% (1/3) 47.7 ± 59.3	28.8 ± 9.3	66.7% (2/3)
Subgroups of focal dystonia						
blepharospasm (*n* = 12)	71.2 ± 16.8	33.3% (4/12) 5.6 ± 4.4	41.7% (5/12) 5.6 ± 4.0	25.0% (3/12) 25.1 ± 28.1	5.4 ± 2.2	25.0% (3/12)
Oromandibular/lingual (*n* = 8)	71.7 ± 15.9	0.0% (0/8) 4.0 ± 3.0	12.5% (1/8) 5.3 ± 2.4	37.5% (3/8) 29.3 ± 15.1	4.2 ± 1.8	0.0% (0/8)
Laryngeal (*n* = 10)	81.5 ± 9.1	10.0% (1/10) 1.8 ± 2.7	50.0% (5/10) 7.3 ± 2.8	40.0% (4/10) 28.3 ± 16.1	3.0 ± 2.1	30.0% (3/10)
Cervical (*n* = 54)	68.2 ± 19.7	25.9% (14/54) 4.6 ± 4.0	48.1% (26/54) 6.8 ± 4.0	40.7% (22/54) 31.3 ± 25.7	4.4 ± 2.2	59.3% (32/54)
Limb (*n* = 9)	73.3 ± 29.8	22.2% (2/9) 3.8 ± 5.0	33.3% (3/9) 6.3 ± 4.6	22.2% (2/9) 15.9 ± 18.7	4.1 ± 2.3	22.2% (2/9)

HR-QoL and BFMDRS: the mean RAND 36-Item Health Survey total score (range 0–100%) and mean BFMDRS score with standard deviation of all patients and per type of dystonia

Depression, GAD, SAD: Percentage and number of patients with symptoms of depression (HADS-D > 7), GAD (HADS-A > 7), SAD (LSAS > 30) and mean HADS-D, mean HADS-A and mean LSAS scores with standard deviation of all patients and per type of dystonia

Tremor: Percentage and number of patients with dystonic tremor of all patients and per type of dystonia

Regarding therapy, 67.1% (104/155) were treated with BoNT, 41.3% (64/155) with oral antidystonic drugs (anticholinergics, benzodiazepines, nonbenzo-hypnotics, dopaminergics, antidopaminergics, muscle relaxants) and 27.7% (43/155) with antidepressants at baseline. None of the patients had a deep brain stimulator (DBS), whereas three patients had previously received a myectomy for blepharospasm or spasmodic dysphonia.

### Predictors of the eight HR-QoL domains within 2 years

Higher depression scores at baseline predicted lower HR-QoL on all eight subscales at 2 years (Total indirect effect: General Health: *β* = – 1.82, Physical Functioning: *β* = – 2.22, Physical Role Functioning: *β* = – 3.41, Pain: *β* = – 1.87, Energy/Fatigue: *β* = – 2.23, Emotional Well-being: *β* = – 1.17, Emotional Role Functioning: *β* = – 3.46, Social Functioning: *β* = – 2.71, all *p* < 0.001). Higher GAD scores at baseline predicted lower HR-QoL related to general health (Total indirect effect: *β* = – 0.87, *p* = 0.005), pain (Total indirect effect: *β* = – 1.50, *p* = 0.002) and emotional well-being (Total indirect effect: *β* = – 1.26, *p* < 0.001) after 2 years, whereas higher SAD scores at baseline predicted higher pain-related QoL (Total indirect effect: *β* = 0.17, *p* = 0.002). Higher dystonia severity at baseline predicted lower HR-QoL in the context of social functioning (Total indirect effect: *β* = – 0.69, *p* = 0.002). The total indirect effects could not be calculated for binary variables such as tremor. Tremor had no direct effects on any of the eight HR-QoL subscales (all *p* > 0.006). The total direct effects of the eight cross-lagged path models are presented in Fig. 1 and Online Resource 2_Fig. 3.

### Predictors of total HR-QoL within 2 years

**Fig. 2:**
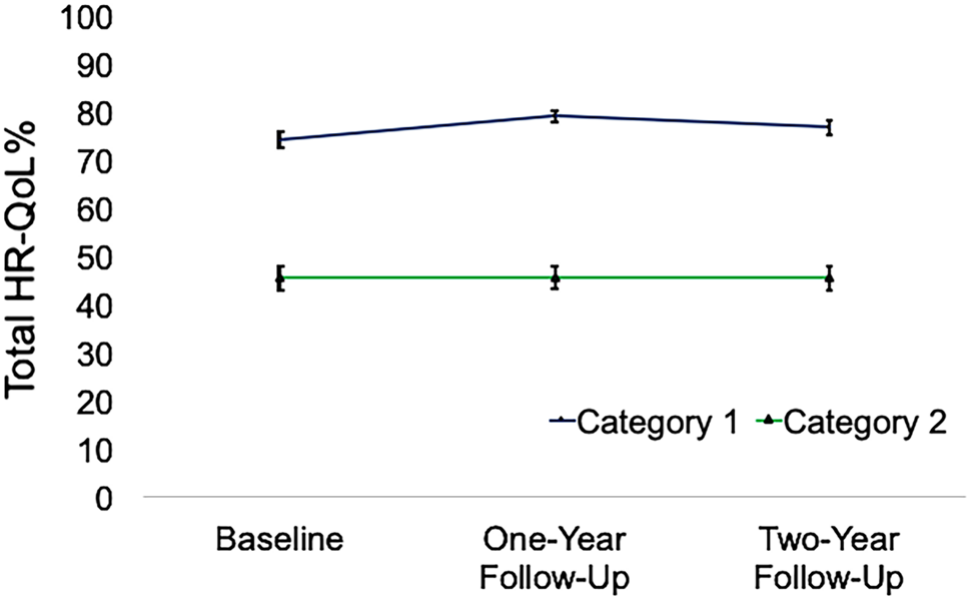
Latent class growth analysis. The LCGA revealed two latent classes (here “categories”), distinguished by the total HR-QoL (in %) that was reported across the three time points (baseline, 1-year follow-up, 2-year follow-up). Category 1 (66% of patients) reported a consistently higher level of HR-QoL that was susceptible to symptoms of depression and SAD, whereas category 2 (34% of patients) reported a consistently lower level of HR-QoL that was susceptible to symptoms of GAD. y-axis represents HR-QoL (total) in percent with standard error

LCGA revealed two categories distinguished by the total HR-QoL that was reported across the three time points by 145 longitudinally examined dystonia patients (Fig. 2).

#### Category 1

66% of patients reported a higher level of quality of life over the three points (mean HR-QoL = 74.36% ± 16.07). In this group, HR-QoL was susceptible to symptoms of depression and SAD. Higher depression scores reported at baseline predicted lower HR-QoL after 2 years (*β* = – 0.40, *p* = 0.047) as did higher levels of SAD (*β* = – 0.42, *p* = 0.017). In contrast, patients with lower depression scores at baseline had a more stable HR-QoL score over the 2-year period (flatter statistical slope, *β* = 0.88, *p* < 0.001). Overall, the patients had no significant increase of HR-QoL trajectories over the 2 years (beta = 1.99, *p* = 0.09). No relationship was present between HR-QoL and severity of GAD (intercept *β* = – 0.37, *p* = 0.095; slope *β* = – 0.29, *p* = 0.32), age (intercept *β* = – 0.33, *p* = 0.093; slope *β* = – 0.29, *p* = 0.203), sex (intercept *β* = – 0.11, *p* = 0.622; slope *β* = – 0.47, *p* = 0.057), or dystonia severity (intercept *β* = 0.01, *p* = 0.972; slope *β* = 0.15, *p* = 0.510).

#### Category 2

34% of patients reported a consistently lower level of HR-QoL over the 2-year period (mean HR-QoL = 45.54% ± 17.64). In this group of patients, HR-QoL was susceptible to symptoms of GAD, i.e., higher GAD baseline levels were associated with lower levels of HR-QoL after 2 years (*β* = – 0.71, *p* < 0.001). There was no relationship between HR-QoL and severity of depression (intercept *β* = – 0.26, *p* = 0.187; slope *β* = 0.79, *p* = 0.190), severity of SAD (intercept *β* = – 0.12, *p* = 0.488; slope *β* = 0.27, *p* = 0.581), age (intercept *β* = – 0.08, *p* = 0.732; slope beta = 0.18, *p* = 0.789), sex (intercept *β* = 0.36, *p* = 0.052, slope *β* = 0.53, *p* = 0.314), or dystonia severity (intercept *β* = 0.13, *p* = 0.520; slope *β* = 0.31, *p* = 0.505). Overall, patients in this class had no significant decrease of HR-QoL trajectories over the 2 years (beta = – 3.78, *p* = 0.331).

### Influence of BoNT therapy on HR-QoL within 1 year

A nonparametric ANOVA with HR-QoL total score as dependent variable, time (1 year) as a within-subject factor and treatment group (BoNT initiated/no BoNT) as a between-subject factor revealed a main effect for time [F(1,67.9) = 9.6, *p* = 0.002] to improve HR-QoL after 1 year, and no effect for group [F(1,67.9) = 1.0, *p* = 0.314] or the interaction between time and group [F(1,67.9) = 0.2, *p* = 0.697]. Another nonparametric ANOVA with BFMDRS as dependent variable, time as a within-subject factor and group as a between-subject factor revealed a main effect for time [F(1,64.3) = 5.5, *p* = 0.019] in the BFMDRS score decline, and no effect for group [F(1,64.3) = 0.1, *p* = 0.738] or the interaction between time and group [F(1,64.3) = 0.04, *p* = 0.846]. Bonferroni corrected alpha was ≤ 0.025.

The results of the nonparametric ANOVAs with the eight HR-QoL subscales (RAND 36-Item Health Survey) as a dependent variable, time as a within-subject factor and group as a between-subject factor are displayed in Table 2. Bonferroni corrected *p* was ≤ 0.006. Group differences were found for emotional well-being only.

**Table 2:** Influence of BoNT therapy on HR-QoL within 1 year

	**Group**	**Time**	Group*Time
General Health (*n* = 72)	[F(1,68.8) = 1.8, p = 0.183]	[F(1,68.8) = 0.1, p = 0.734]	[F(1,68.8) = 0.3, p = 0.610]
Physical Functioning (*n* = 72)	[F(1,69.9) = 0.4, p = 0.511]	[F(1,69.9) = 1.2, p = 0.267]	[F(1,69.9) = 0.3, p = 0.576]
Physical Role Functioning (*n* = 72)	[F(1,69.3) = 0.01, p = 0.928]	[F(1,69.3) = 6.5, p = 0.011]	[F(1,69.3) = 0.4, p = 0.542]
Pain (*n* = 71)	[F(1,68.0) = 0.0005, p = 0.983]	[F(1,68.0) = 0.3, p = 0.562]	F(1,68.0) = 1.9, p = 0.173]
Energy/ Fatique (*n* = 72)	[F(1,70.0) = 0.3, p = 0.601]	[F(1,70.0) = 6.4, *p* = *0.012*]	[F(1,70.0) = 0.6, p = 0.455]
Emotional Well-being (*n* = 72)	[F(1,70.0) = 8.2, **p = 0.004**]	[F(1,70.0) = 5.3, *p* = *0.022*]	[F(1,70.0) = 0.4, p = 0.545]
Emotional Role Functioning (*n* = 71)	[F(1,64.4) = 1.1, p = 0.299]	[F(1,64.4) = 3.3, p = 0.071]	[F(1,64.4) = 0.1, p = 0.786]
Social Functioning (*n* = 72)	[F(1,69.0) = 4.5, *p* = *0.034*]	[F(1,69.0) = 5.3, *p* = *0.021*]	[F(1,69.0) = 0.2, p = 0.684]

Nonparametric ANOVAs with the 8 HR-QoL subscales (RAND 36-Item Health Survey) as dependent variable, time (1 year) as a within-subjects factor and group as a between-subjects factor and interactions between group*time. Bonferroni corrected alpha ≤ 0.006

The BoNT-treated group had higher depression scores (HADS-D) at baseline than the untreated group (*U* = 360.0, *p* = 0.002), whereas anxiety (HADS-A; *U* = 470.0, *p* = 0.066) and social anxiety scores (LSAS; *U* = 446.5, *p* = 0.036) did not differ between groups (Bonferroni corrected alpha was ≤ 0.017, Table 3). Age (*U* = 583.0, *p* = 0.467) and sex (*χ*2 = 0.498; *p* = 0.633) were not different between the two groups.

**Table 3:** Clinical data of the patients with and without BoNT treatment at baseline

	**HR-QoL**	**Depression**	**Generalized anxiety**	**Social anxiety**	**BFMDRS**	**Tremor**
patients without BoNT (*n* = 37)	69.0 ± 19.7	10.8% (4/37) 3.0 ± 3.1	27% (10/37) 5.3 ± 3.8	43.2% (16/37) 29.6 ± 25.7	6.3 ± 4.4	43.2% (16/37)
patients with BoNT (*n* = 35)	63.4 ± 22.2	28.6% (10/35) 5.6 ± 4.1	34.3% (12/35) 7.0 ± 4.3	60.0% (21/35) 43.4 ± 29.9	6.7 ± 4.8	31.4% (11/35)

HR-QoL and BFMDRS: Mean RAND 36-Item Health Survey total score (range 0–100%) and mean BFMDRS score with standard deviation for patients with and without BoNT treatment

Depression, GAD, SAD: Percentage and number of patients with symptoms of depression (HADS-D > 7), GAD (HADS-A > 7), SAD (LSAS > 30) and mean HADS-D, mean HADS-A and mean LSAS scores with standard deviation for patients with and without BoNT treatment

Tremor: Percentage and number of patients with dystonic tremor for patients with and without BoNT treatment

## Discussion

Our international, multicenter study aimed to identify predictors of HR-QoL in isolated dystonia involving 155 patients over a time period of 2 years. Symptoms of depression predicted all eight subdomains of HR-QoL, whereas higher generalized anxiety scores predicted lower HR-QoL of three subdomains (general health, pain, emotional well-being) and higher social anxiety symptoms higher pain-related QoL. In contrast, dystonia severity was associated with limitations in social functioning only. We additionally separated two categories of patients with either higher or lower HR-QoL and identified a distinct profile of predictors in each. HR-QoL was more dependent on symptoms of depression and social anxiety in patients with a higher QoL whereas generalized anxiety predicted HR-QoL in the group of patients with a lower HR-QoL.

Symptoms of depression are a strong determinator of HR-QoL in dystonia which is in keeping with results of several previous cross-sectional studies in cervical and craniofacial dystonia [3, 9, 12] and other forms of isolated dystonia [8, 30, 31]. In our previous cross-sectional study involving 603 patients from the same cohort including the patients investigated here, and in a study in cervical dystonia, also symptoms of GAD and SAD were associated with all eight subdomains of HR-QoL [8, 9]. Interestingly, this effect was not as strong when the patients in the present study were investigated longitudinally, as fewer domains appeared to be influenced by symptoms of GAD (*n* = 3) and SAD (*n* = 1). This could be in part explained by the smaller sample size leading to less statistical power. However, a recent cross-sectional study in patients with craniofacial dystonia showed similar results using multiple linear regression analysis of depression, anxiety, and SF-36 scores. Anxiety correlated with all eight subdomains of SF36, but after adjusting for disease duration, education level, and depression correlated only with the pain subdomain [12]. Although depression predicted all subdomains of future HR-QoL in our study, GAD predicted less but relevant subdomains such as general health, emotional well-being and pain and GAD was associated with a significantly lower total QoL as compared to depression (mean HR-QoL: 45.5% ± 17.6 vs. 74.4% ± 16.1). This may in part be due to behavioral and pharmacologic therapy for GAD being less frequently employed as compared to the established guidelines of depression treatment. This highlights the importance of screening for symptoms of both psychiatric disorders. Of note, higher SAD scores predicted higher pain-related QoL at 2 years, which was unexpected. A possible interpretation of this association is, however, that patients with SAD may be less active, resulting in reduced bodily activity, which may consequently be associated with less occurrence of pain. Furthermore, there might be a pathway convergence of the anxiety and medial nociceptive system, which is connected to the cingulate gyrus, amygdala and hypothalamus, sub-serving the affective-motivational aspects of pain [32, 33].

Dystonia severity was a determinant of HR-QoL in cervical dystonia and was associated with four out of eight subdomains of HR-QoL in our cross-sectional study analysis [8, 9]. The effect vanished, however, in the longitudinal design of the present study with dystonia severity only being a predictor in the context of social functioning. The high burden on HR-QoL of NMS rather than solely MS of dystonia, as also proven e.g. in patients with writer’s cramp [34], thus emphasizes the impact of coping strategies, acceptance of illness, as well as management of mood and anxiety on the maintenance of an acceptable HR-QoL [10]. Nevertheless, methodological differences, such as dystonia severity being rated by patients [9] or movement disorder specialists [8], as well as the use of different rating scales, should be taken into account. The BFMDRS, which is a validated [25] and commonly used rating scale, can be limited when assessing different focal dystonia phenotypes. In our study, 93 patients had focal dystonia and only 40% segmental, multifocal or generalized dystonia. Furthermore, 67% of our patients were treated with BoNT about three months prior to the examination. Thus, dystonia severity may have been potentially higher in some patients without long-term BoNT therapy [35] and may have resulted in stronger associations with lower HR-QoL. This question could only be answered with a longitudinal study design in which a large number of untreated and significantly affected dystonia patients were included and BoNT therapy was initiated during the course of the study to evaluate the effect at the time of peak BoNT efficacy.

In contrast to our previous cross-sectional results on HR-QoL in dystonia showing tremor impacting HR-QoL in the context of physical functioning and pain [8], here tremor was not found to predict future HR-QoL. As in our cross-sectional study, only the existence of tremor, but not its severity or distribution, was evaluated which is a limitation [8]. This still highlights that NMS have a greater impact on the quality of life of dystonia patients than MS. Nevertheless, a long-term improvement in patients with long-term BoNT therapy cannot be excluded, although patients were evaluated about three months after BoNT treatment.

Interestingly, there was a similar 1-year improvement in dystonia severity and total HR-QoL in a subgroup of patients with and without BoNT therapy. There were no group differences regarding age, sex and HR-QoL subscales except for emotional well-being, which was most likely a result of higher baseline depression scores in the BoNT-treated group. These results are in contrast to recently published data describing unchanged total QoL in patients with cervical dystonia at 2 years. However, these results are not comparable because only patients with cervical dystonia were included and the assessment was performed approximately 12 weeks after the previous BoNT injection which is different from our design [21]. Furthermore, the CDIP‐58, used by Moriarty et al. [21]**,** is a cervical dystonia-specific patient self-report of QoL [36] with a stronger focus on motor symptoms as compared to the RAND 36, a commonly used, generic quality of life questionnaire [37]. We can only speculate as to the significance of the finding of a similar 1-year improvement in dystonia severity and total HR-QoL independent of the BoNT effect. Several possibilities are proposed. First, there may be a nonspecific quality-enhancing effect created by the close clinical monitoring required in a research protocol, expanding into those not receiving BoNT. Furthermore, the factors unrelated to those targeted by chemodenervation and unaccounted for in this study may have yielded the magnitude of effects quantified by the HR-QoL [10]. Physical and cognitive − behavioral therapy has been shown to improve dystonia severity and HR-QoL in cervical dystonia [38–42]. These aspects, however, were not systematically assessed here. Alternatively, the beneficial effects of BoNT may not be readily captured by the evaluation of HR-QoL at the end of the BoNT treatment cycle resulting in the absence of a significant interaction between time and group. Examinations in cranial and cervical dystonia four, six or eight weeks after BoNT injection likewise revealed an improvement of dystonia severity and improvement of some HR-QoL domains [3, 15–19]. In patients with cervical dystonia, nonmotor symptoms as depression and anxiety improved one month after BoNT treatment [3, 15, 43] with a slighter but still significant improvement after three months [43]. Thus, the decreased effect of BoNT at the time point of the follow-up visit of the present study is likely to have an impact on the results of measurement in the patient group with BoNT treatment.

This longitudinal study has some limitations: To guarantee statistical power some potential confounding factors such as educational level, marital and socio-economic status were not assessed but may significantly influence HR-QoL. The BFMDRS is a validated [25] and commonly used rating scale for dystonia. Nevertheless, it may not be sufficiently representative of focal dystonias. Furthermore, only the existence and not the severity, distribution, frequency, amplitude and regularity of tremor were evaluated. Examination of patients at the end of the BoNT treatment cycle only allowed the evaluation of long-term effects. In addition, samples sizes of this subanalysis were not large enough to detect significant small effects (at *α* = 0.05). However, the groups with and without BoNT treatment were well defined and well corrected for other therapeutic influences.

In summary, the most comprehensive predictors of HR-QoL in isolated dystonia are symptoms of depression, followed by GAD, whereas severity of dystonia only predicts social functioning. Dystonia patients with higher levels of anxiety have lower HR-QoL than patients with symptoms of depression and social anxiety, possibly as a consequence of less utilization of medication and behavioral therapy for GAD. Although standard therapy regimens as BoNT mainly focus on the physical symptoms, depression and anxiety should be specifically identified and targeted to improve long-term HR-QoL in dystonia.

## Standard Protocol Approvals and Patient Consents

All participants gave written informed consent for study participation prior to their enrollment. The study was approved by the local ethics committees of all clinical sites and performed in accordance with the ethical standards laid down in the 1964 Declaration of Helsinki and its later amendments.

## Supplementary Information

Below is the link to the electronic supplementary material.

**Online Resource 1.** Coinvestigators of the Dystonia Coalition Study Group, that had a major role in the acquisition of data 1 (PDF 115 kb)

**Online Resource 2.** Direct effects of the cross-lagged path model (stylized illustration). Direct paths of the cross-lagged path model for the physical functioning, physical role functioning, pain, energy / fatigue, emotional well-being, emotional role functioning and the social functioning subscales of HR-QoL are presented. Dashed lines illustrate the effect of age and sex on the different variables. Bonferroni corrected alpha is ≤ 0.006. Significant paths are marked in red, grey paths did not reach significance level 2 (PDF 686 kb)
