# Supplementary material for: Longitudinal predictors of health-related quality of life in isolated dystonia
Source: J Neurol. 2023 Oct 15;271(2):852–63. doi: 10.1007/s00415-023-12022-4 (PMC10827910; doi:10.1007/s00415-023-12022-4)
Supplement: Supplementary file 1 — Online Resource 1. Coinvestigators of the Dystonia Coalition Study Group, that had a major role in the acquisition of data 1 (PDF 115 kb) [file 415_2023_12022_MOESM1_ESM.pdf]

Longitudinal predictors of health-related quality of life in isolated dystonia

Johanna Junker, MD[1,2], James Hall, DPhil[3], Brian D. Berman, MD, MS[4], Marie Vidailhet, MD[5,6], Emmanuel Roze, MD, PhD[5], Tobias Bäumer, MD[7], Irene A. Malaty, MD[8], Aparna Wagle Shukla, MD[8], Joseph Jankovic, MD[9], Stephen G. Reich, MD[10], Alberto J. Espay, MD[11], Kevin R. Duque, MD[11], Neepa Patel, MD[12], Joel S. Perlmutter, MD[13], H. A. Jinnah, MD, PhD[14], Dystonia Coalition Study Group[15], Valerie Brandt, PhD*[16], Norbert Brüggemann, MD*[1,2]

[1] Institute of Neurogenetics, University of Luebeck, Luebeck, Germany
[2] Department of Neurology, University of Luebeck, Luebeck, Germany
[3] Southampton Education School, University of Southampton, UK
[4] Department of Neurology, Virginia Commonwealth University, Richmond, VA, USA
[5] AP-HP, Hopital de la Pitie-Salpetriere, Departement de Neurologie, Paris, France
[6] Sorbonne Université, Institut du Cerveau_ Paris Brain Institute-ICM, INSERM 1127, CNRS 7225, Paris, France
[7] Institute of Systems Motor Science, University of Luebeck, Luebeck, Germany
[8] Department of Neurology, Fixel Institute for Neurologic Disorders, University of Florida, Gainesville, Florida, USA
[9] Parkinson's Disease Center and Movement Disorders Clinic, Department of Neurology, Baylor College of Medicine, Houston, Texas, USA
[10] Department of Neurology, University of Maryland, School of Medicine, Baltimore, MD, USA
[11] Department of Neurology, University of Cincinnati, Cincinnati, OH, USA
[12] RUSH Parkinson's disease and Movement Disorders Center, Department of Neurological Science, RUSH University Medical Center Chicago, Chicago, IL, USA
[13] Departments of Neurology, Radiology & Neuroscience, Washington University in St. Louis, St. Louis, MO, USA
[14] Department of Neurology and Human Genetics, Emory University, Atlanta, GA, USA
[15] see Supplement 1
[16] School of Psychology, Centre for Innovation in Mental Health, University of Southampton, UK

*These authors have contributed equally to the work

Journal name: Journal of Neurology

Corresponding Author:
Norbert Brüggemann, MD
Dept. of Neurology and Institute of Neurogenetics, University of Lübeck
Ratzeburger Allee 160
Lübeck, SH, 23538, Germany
Phone +49-451-500 43400
Fax +49-451-500 43404
norbert.brueggemann@uni-luebeck.de
https://orcid.org/0000-0001-5969-6899

Supplement 1

Coinvestigators of the Dystonia Coalition Study Group, that had a major role in the acquisition of data:

Name	Affiliations
Agarwal P., MD, FAAN	Booth Gardner Parkinson Care Center (Kirkland, WA, USA)
Barbano R. L., MD, PhD	University of Rochester (Rochester, NY, USA)
Berardelli A., MD	Universitá Degli Studi Di Roma „La Sapienza“ (Rome, Italy)
Berman B., MD	University of Colorado (Aurora, CO, USA)
Brashear A., MD	Wake Forest Health Sciences (Winston-Salem, NC, USA)
Chouinard S., MD	CHUM, University of Montreal (Montreal, Quebec, Canada)
Comella C. L., MD	Rush University (Chicago, IL, USA)
Espay A., MD	University of Cincinnati (Cincinnati, OH, USA)
Fox S. H., MD, PhD	Toronto Western Hospital (Toronto, Ontario, Canada)
Fung V. S. C., MD	Westmead Hospital (Westmead, New South Wales, Australia)
Grill S., MD, PhD	Parkinsons and Movement Disorders Center of Maryland (Elkridge, MD, USA)
Hallett M., MD	National Institutes of Health (Bethesda, Maryland, USA)
Harlow T. L., MD	Sanford Health - Fargo (Fargo, ND, USA)
Jankovic J., MD	Baylor College of Medicine (Houston, TX, USA)
Jinnah H. A., MD, PhD	Emory University (Atlanta, GA, USA)
Khemani P., MD<br>Patel, N., MD	University of Texas Southwestern (Dallas, TX, USA)
Klein C., MD	University of Luebeck (Luebeck, Germany)
LeDoux M. S., MD, PhD	University of Tennessee (Memphis, TN, USA)
Leegwater-Kim J., MD, PhD	Lahey Clinic (Burlington, MA, USA)
Malaty I., MD	University of Florida (Gainesville, FL, USA)
Mari Z. K., MD	Johns Hopkins University (Baltimore, MD, USA)

Nahab, F. B., MD	University of California San Diego (La Jolla, CA, USA)
Ondo W. G., MD	Methodist Hospital Research Institute (Houston, TX, USA)<br>University of Texas (Austin, TX, USA)
Perlmutter J., MD	Washington University in St. Louis (St. Louis, MO, USA)
Reich S., MD	University of Maryland (Baltimore, MD, USA)
Richardson S. P., MD	University of New Mexico (Albuquerque, NM, USA)
Severt L., MD	Beth Israel Medical Center (New York, NY, USA)
Shih L., MD	Beth Israel Deaconess (Boston, MA, USA)
Stover N., MD	University of Alabama (Tuscaloosa, AL, USA)
Suchowersky O., MD	University of Alberta (Edmonton, Alberta, Canada)
Testa C. M., MD, PhD	Virginia Commonwealth University (Richmond, VA, USA)
Truong D., MD	Parkinson`s and Movement Disorders Institute (Fountain Valley, CA, USA)
Uc, E. Y., MD	University of Iowa (Iowa City, IA, USA)
Vidaihet M., MD;<br>Flamand-Roze E., MD	Hospital de la Salpetriere (Paris, France)
Xie T., MD, PhD	University of Chicago (Chicago, IL, USA)
