# Supplementary material for: Longitudinal predictors of health-related quality of life in isolated dystonia
Source: J Neurol. 2023 Oct 15;271(2):852–63. doi: 10.1007/s00415-023-12022-4 (PMC10827910; doi:10.1007/s00415-023-12022-4)
Supplement: Supplementary file 2 — Online Resource 2. Direct effects of the cross-lagged path model (stylized illustration). Direct paths of the cross-lagged path model for the physical functioning, physical role functioning, pain, energy / fatigue, emotional well-being, emotional role functioning and the social functioning subscales of HR-QoL are presented. Dashed lines illustrate the effect of age and sex on the different variables. Bonferroni corrected alpha is ≤ 0.006. Significant paths are marked in red, grey paths did not reach significance level 2 (PDF 686 kb) [file 415_2023_12022_MOESM2_ESM.pdf]

Longitudinal predictors of health-related quality of life in isolated dystonia

Johanna Junker, MD[1,2], James Hall, DPhil[3], Brian D. Berman, MD, MS[4], Marie Vidailhet, MD[5,6], Emmanuel Roze, MD, PhD[5], Tobias Bäumer, MD[7], Irene A. Malaty, MD[8], Aparna Wagle Shukla, MD[8], Joseph Jankovic, MD[9], Stephen G. Reich, MD[10], Alberto J. Espay, MD[11], Kevin R. Duque, MD[11], Neepa Patel, MD[12], Joel S. Perlmutter, MD[13], H. A. Jinnah, MD, PhD[14], Dystonia Coalition Study Group[15], Valerie Brandt, PhD*[16], Norbert Brüggemann, MD*[1,2]

[1] Institute of Neurogenetics, University of Luebeck, Luebeck, Germany
[2] Department of Neurology, University of Luebeck, Luebeck, Germany
[3] Southampton Education School, University of Southampton, UK
[4] Department of Neurology, Virginia Commonwealth University, Richmond, VA, USA
[5] AP-HP, Hopital de la Pitie-Salpetriere, Departement de Neurologie, Paris, France
[6] Sorbonne Université, Institut du Cerveau_ Paris Brain Institute-ICM, INSERM 1127, CNRS 7225, Paris, France
[7] Institute of Systems Motor Science, University of Luebeck, Luebeck, Germany
[8] Department of Neurology, Fixel Institute for Neurologic Disorders, University of Florida, Gainesville, Florida, USA
[9] Parkinson's Disease Center and Movement Disorders Clinic, Department of Neurology, Baylor College of Medicine, Houston, Texas, USA
[10] Department of Neurology, University of Maryland, School of Medicine, Baltimore, MD, USA
[11] Department of Neurology, University of Cincinnati, Cincinnati, OH, USA
[12] RUSH Parkinson's disease and Movement Disorders Center, Department of Neurological Science, RUSH University Medical Center Chicago, Chicago, IL, USA
[13] Departments of Neurology, Radiology & Neuroscience, Washington University in St. Louis, St. Louis, MO, USA
[14] Department of Neurology and Human Genetics, Emory University, Atlanta, GA, USA
[15] see Supplement 1
[16] School of Psychology, Centre for Innovation in Mental Health, University of Southampton, UK

*These authors have contributed equally to the work

Journal name: Journal of Neurology

Corresponding Author:
Norbert Brüggemann, MD
Dept. of Neurology and Institute of Neurogenetics, University of Lübeck
Ratzeburger Allee 160
Lübeck, SH, 23538, Germany
Phone +49-451-500 43400
Fax +49-451-500 43404
norbert.brueggemann@uni-luebeck.de
https://orcid.org/0000-0001-5969-6899

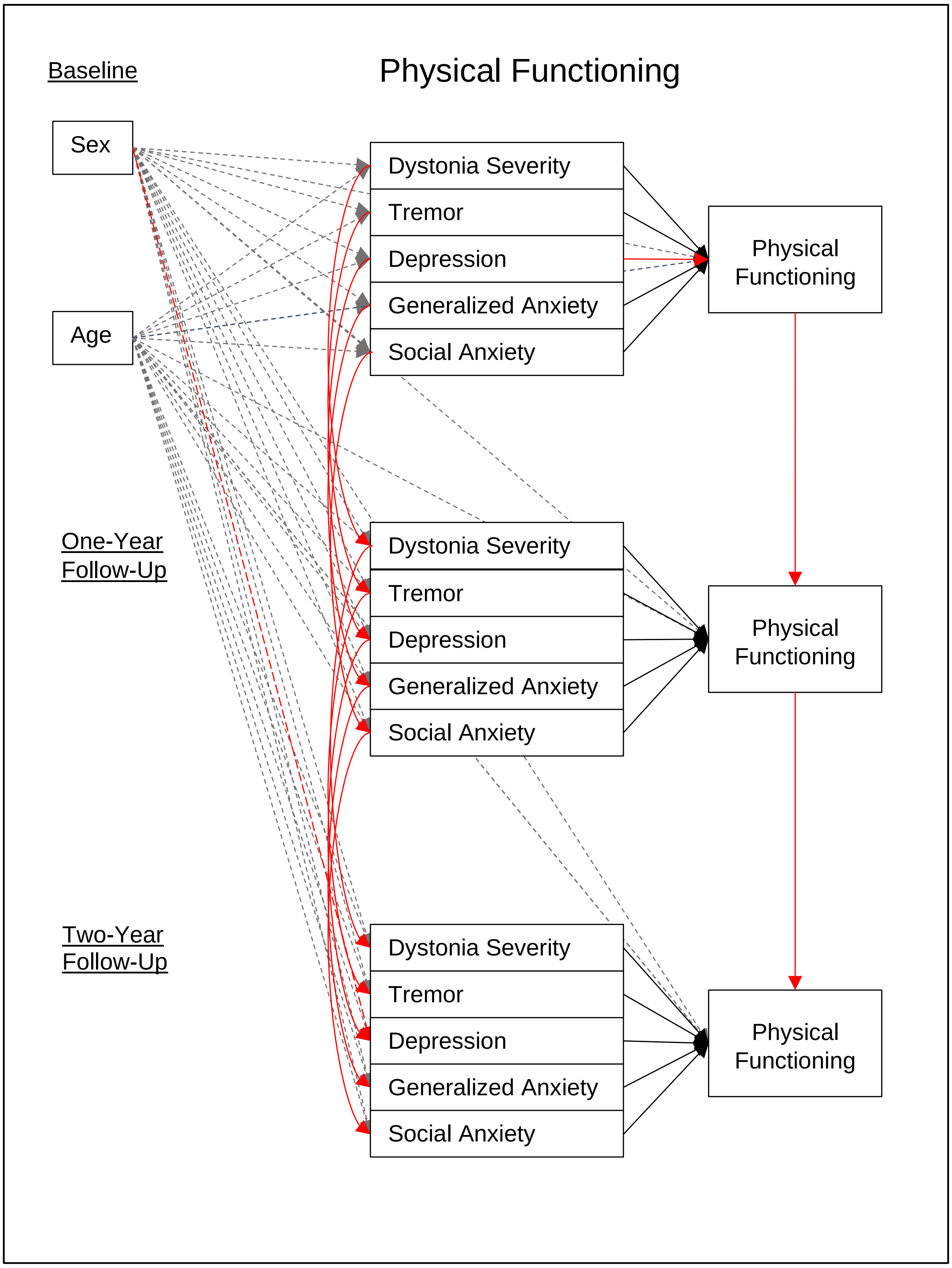

Baseline
Physical Functioning
Sex
Age
Dystonia Severity
Tremor
Depression
Generalized Anxiety
Social Anxiety
Physical Functioning
One-Year Follow-Up
Dystonia Severity
Tremor
Depression
Generalized Anxiety
Social Anxiety
Physical Functioning
Two-Year Follow-Up
Dystonia Severity
Tremor
Depression
Generalized Anxiety
Social Anxiety
Physical Functioning

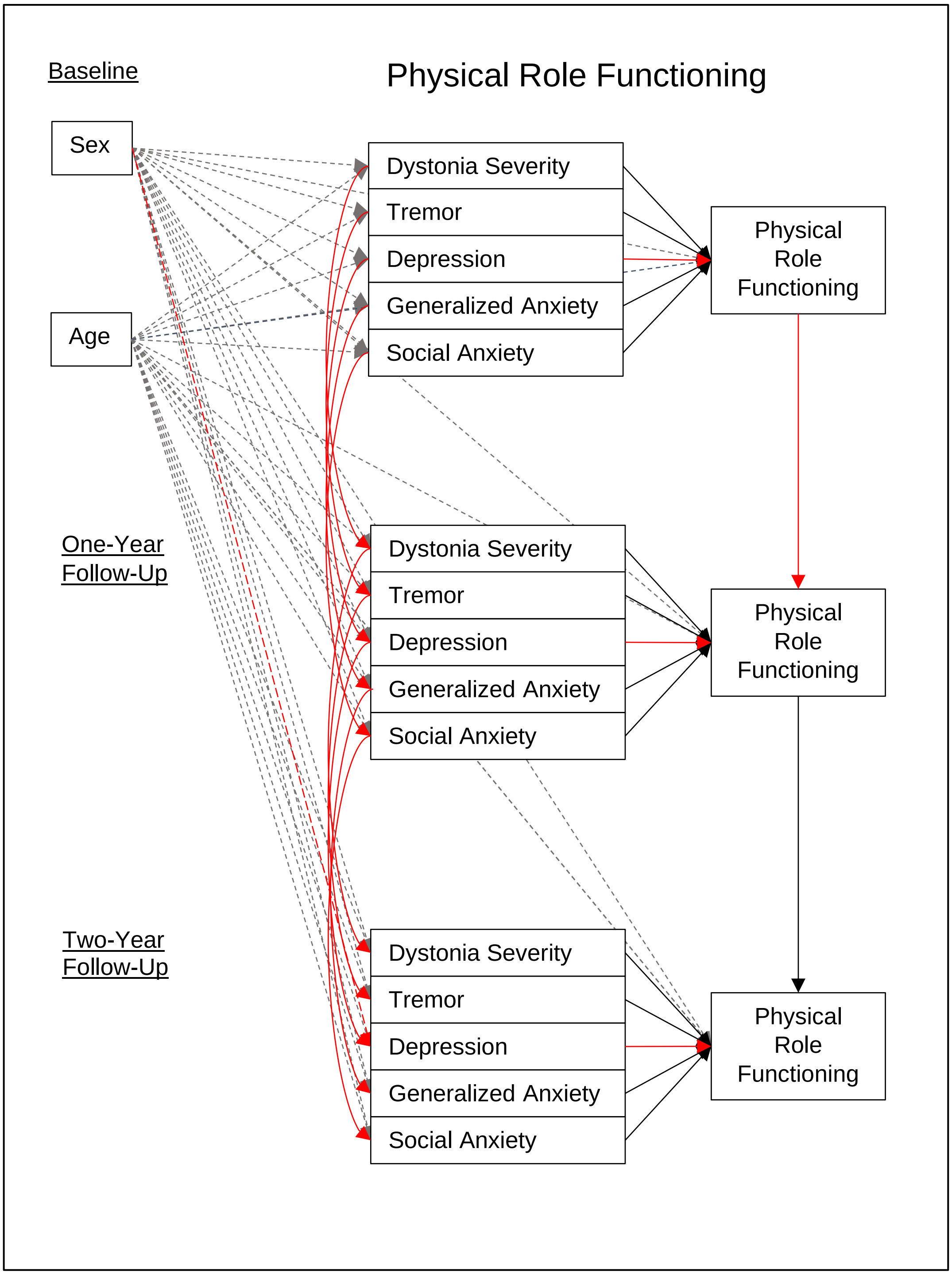
Baseline
Physical Role Functioning
Sex
Age
Dystonia Severity
Tremor
Depression
Generalized Anxiety
Social Anxiety
Physical Role Functioning
One-Year Follow-Up
Dystonia Severity
Tremor
Depression
Generalized Anxiety
Social Anxiety
Physical Role Functioning
Two-Year Follow-Up
Dystonia Severity
Tremor
Depression
Generalized Anxiety
Social Anxiety
Physical Role Functioning

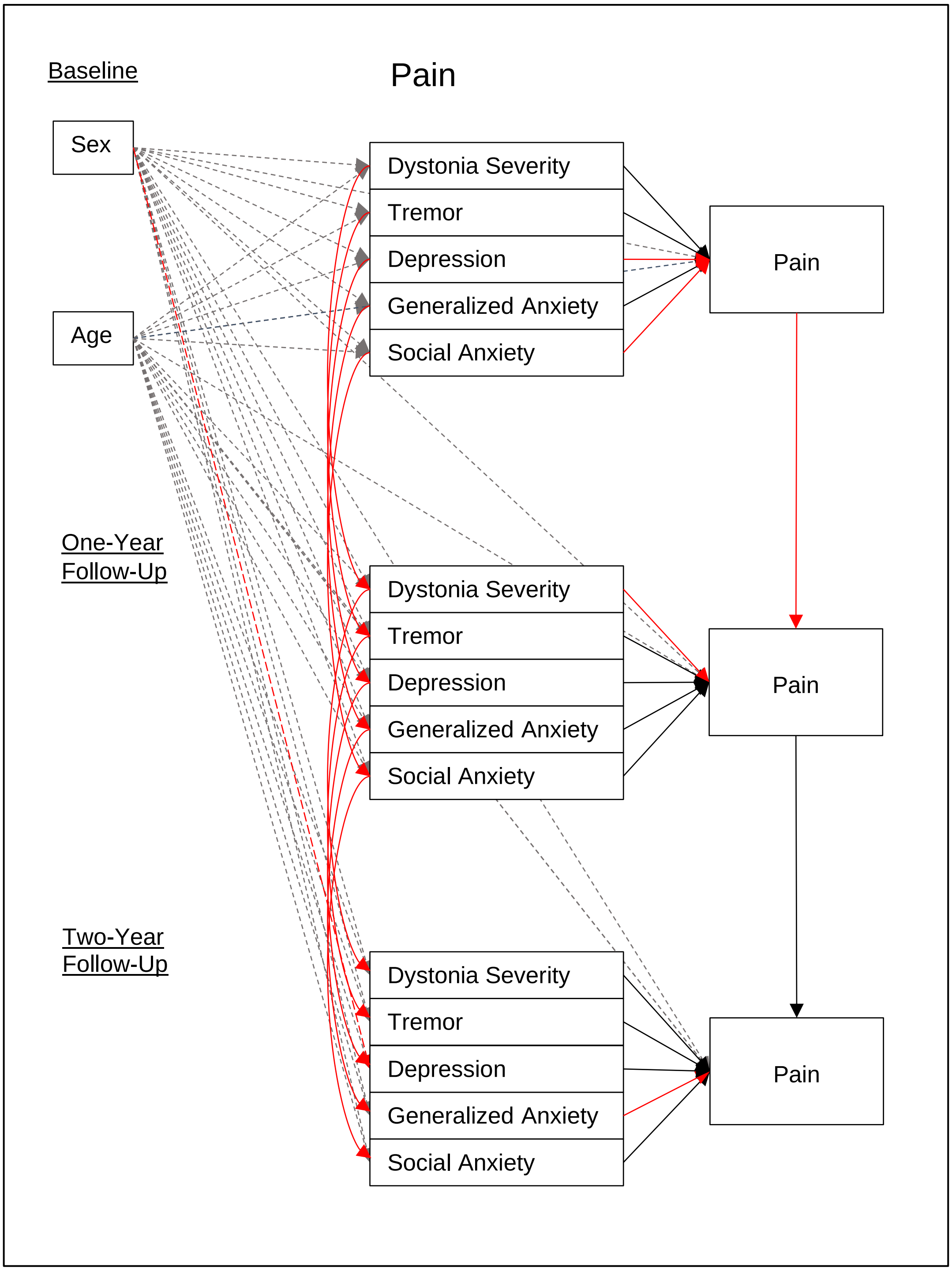
Pain
Baseline
Sex
Age
Dystonia Severity
Tremor
Depression
Generalized Anxiety
Social Anxiety
Pain
One-Year
Follow-Up
Dystonia Severity
Tremor
Depression
Generalized Anxiety
Social Anxiety
Pain
Two-Year
Follow-Up
Dystonia Severity
Tremor
Depression
Generalized Anxiety
Social Anxiety
Pain

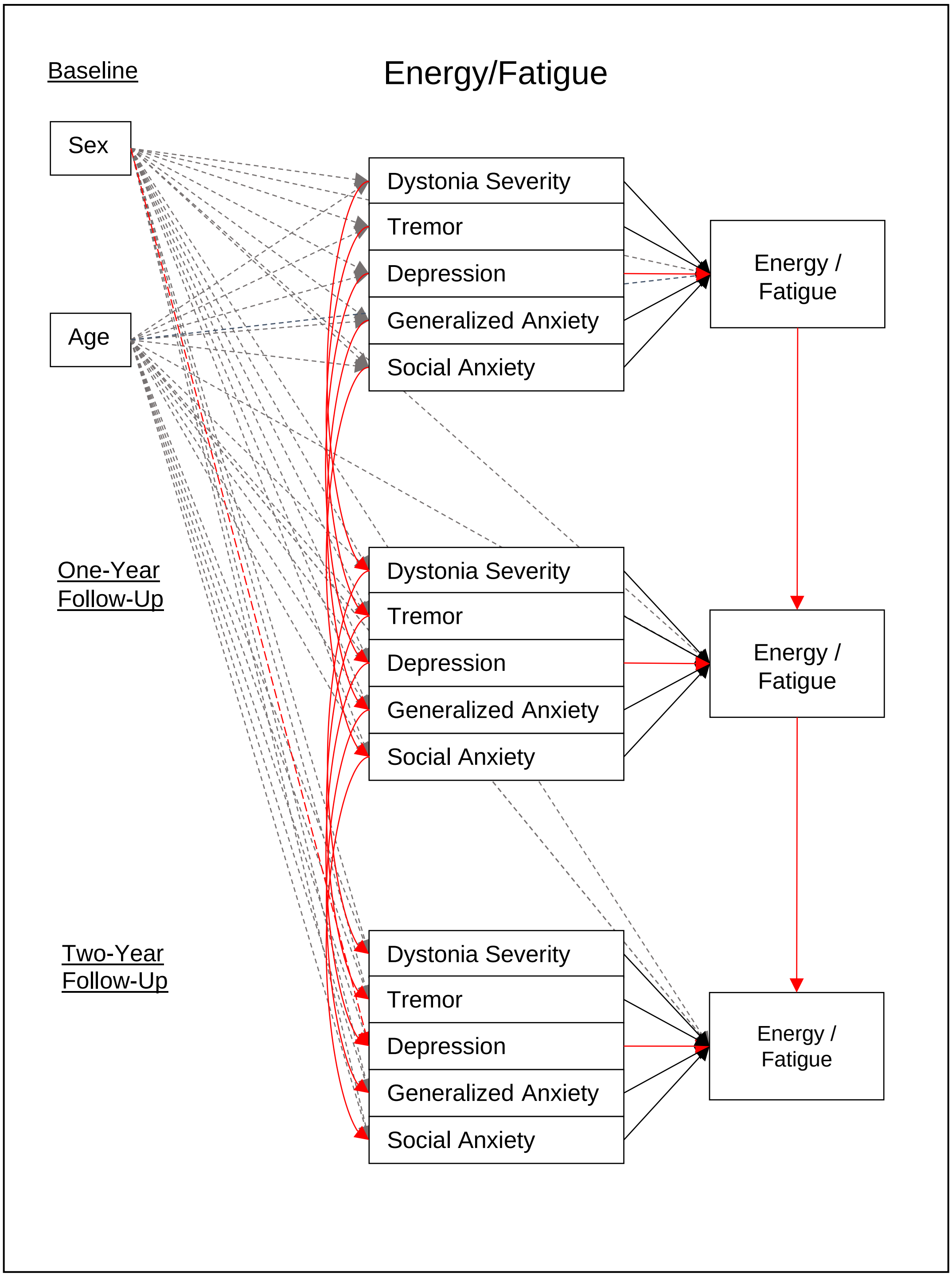

Baseline
Energy/Fatigue
Sex
Age
Dystonia Severity
Tremor
Depression
Generalized Anxiety
Social Anxiety
Energy / Fatigue
One-Year Follow-Up
Dystonia Severity
Tremor
Depression
Generalized Anxiety
Social Anxiety
Energy / Fatigue
Two-Year Follow-Up
Dystonia Severity
Tremor
Depression
Generalized Anxiety
Social Anxiety
Energy / Fatigue

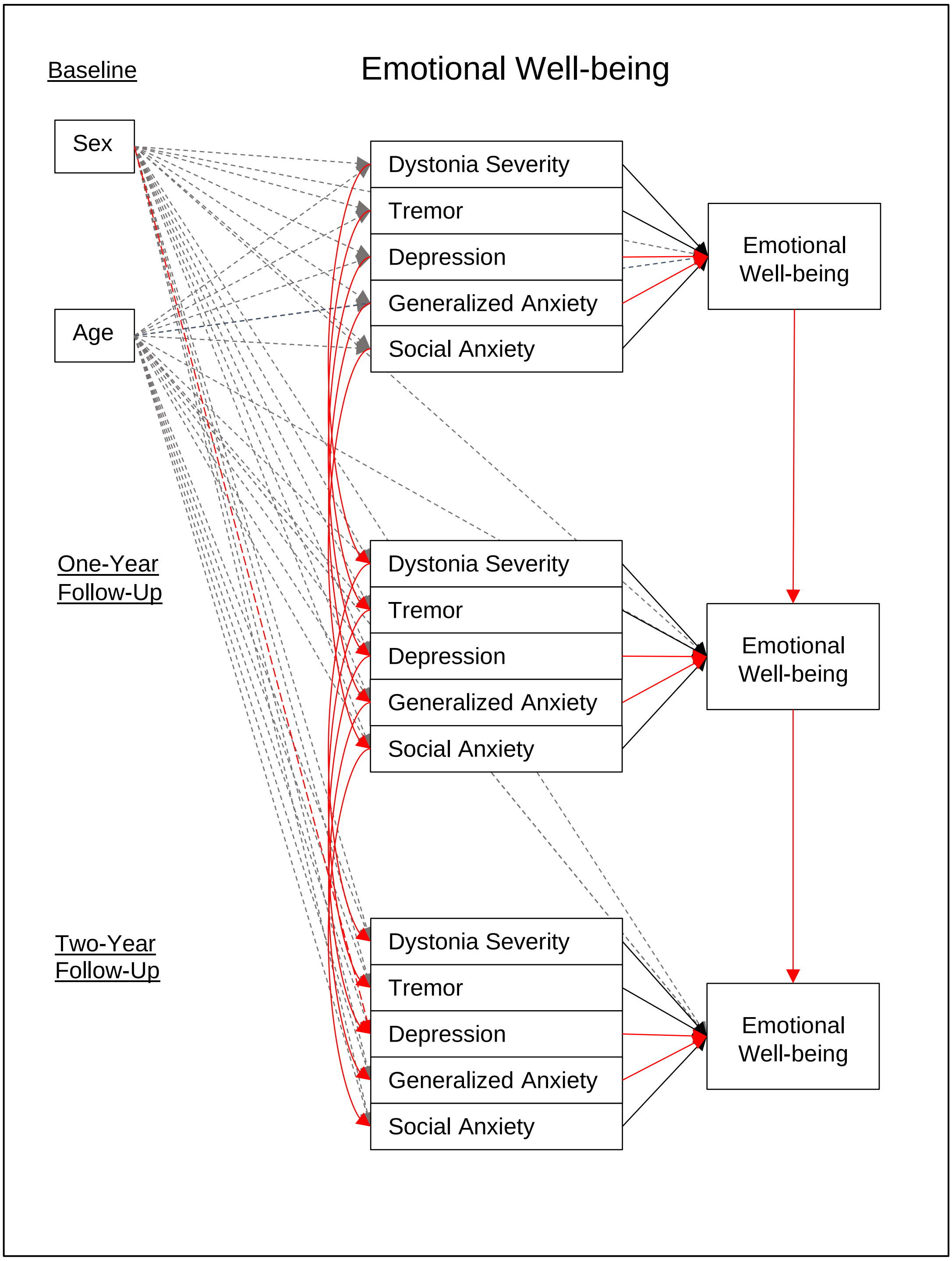
Baseline
Emotional Well-being
Sex
Age
Dystonia Severity
Tremor
Depression
Generalized Anxiety
Social Anxiety
Emotional Well-being
One-Year Follow-Up
Dystonia Severity
Tremor
Depression
Generalized Anxiety
Social Anxiety
Emotional Well-being
Two-Year Follow-Up
Dystonia Severity
Tremor
Depression
Generalized Anxiety
Social Anxiety
Emotional Well-being

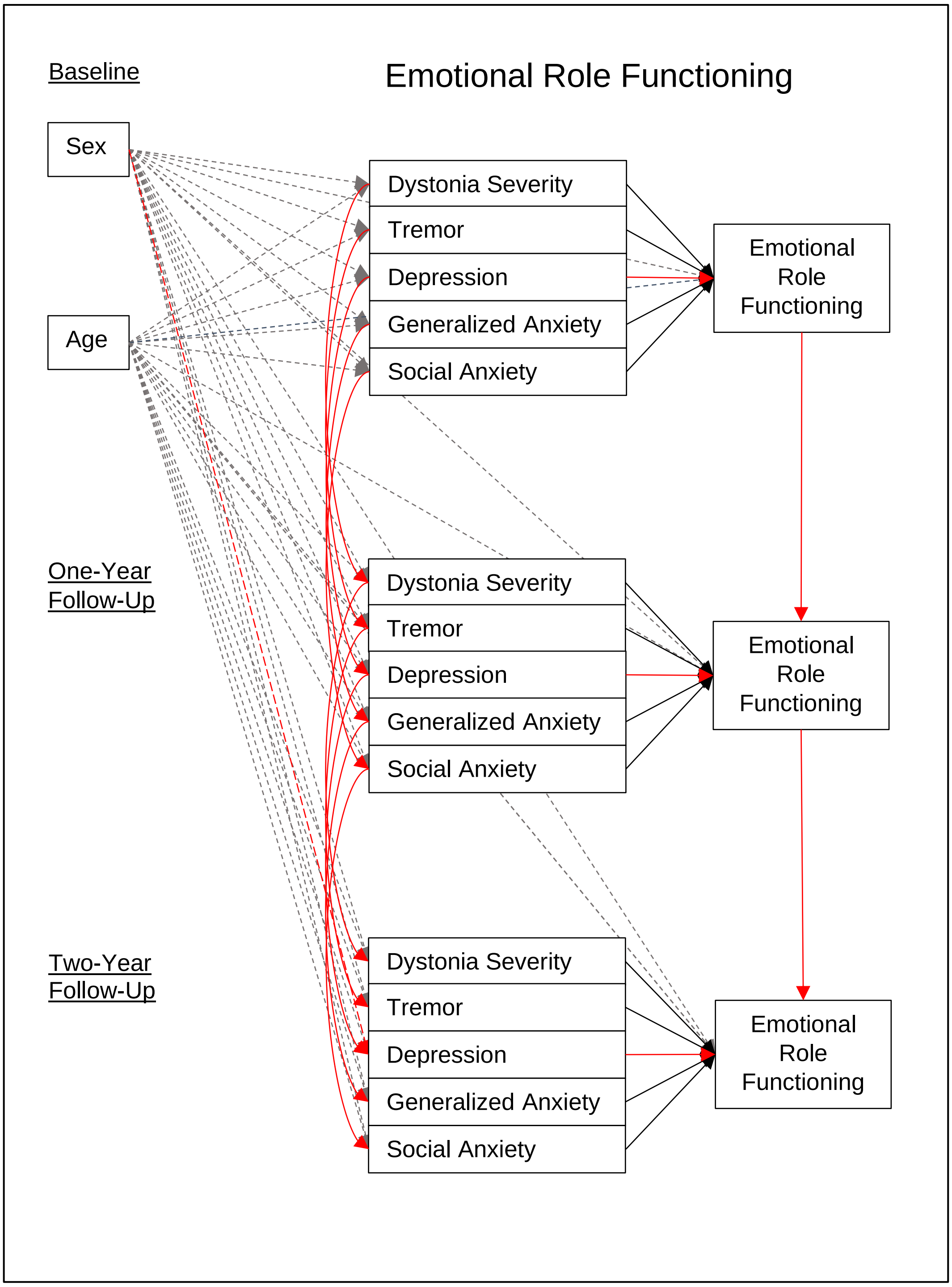

Baseline
Emotional Role Functioning
Sex
Age
Dystonia Severity
Tremor
Depression
Generalized Anxiety
Social Anxiety
Emotional Role Functioning
One-Year Follow-Up
Dystonia Severity
Tremor
Depression
Generalized Anxiety
Social Anxiety
Emotional Role Functioning
Two-Year Follow-Up
Dystonia Severity
Tremor
Depression
Generalized Anxiety
Social Anxiety
Emotional Role Functioning

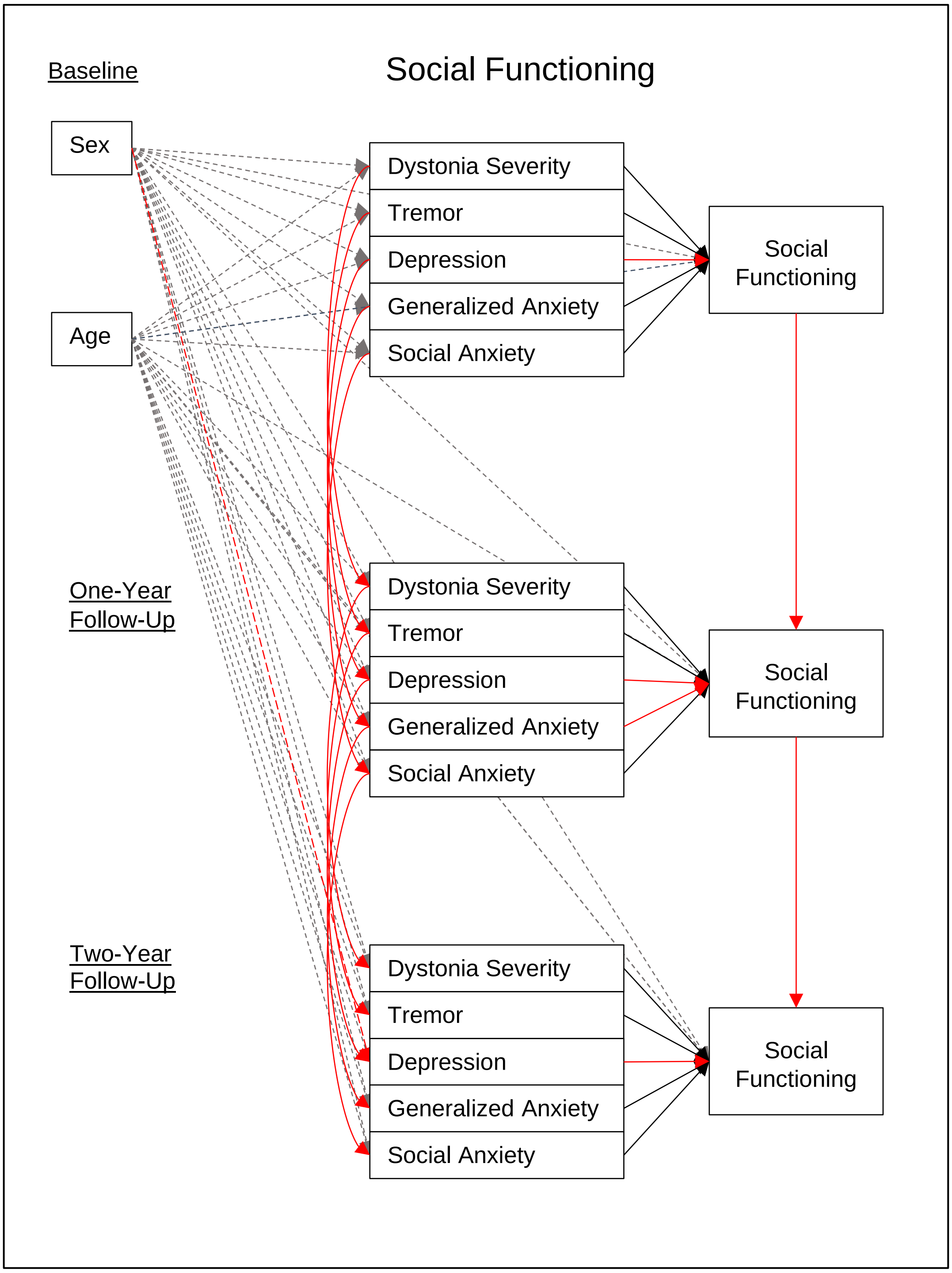
Baseline
Social Functioning
Sex
Age
Dystonia Severity
Tremor
Depression
Generalized Anxiety
Social Anxiety
Social Functioning
One-Year Follow-Up
Dystonia Severity
Tremor
Depression
Generalized Anxiety
Social Anxiety
Social Functioning
Two-Year Follow-Up
Dystonia Severity
Tremor
Depression
Generalized Anxiety
Social Anxiety
Social Functioning
